# Research on ACEI of Low-Molecular-Weight Peptides from *Hirudo nipponia* Whitman

**DOI:** 10.3390/molecules27175421

**Published:** 2022-08-24

**Authors:** Zhao Ding, Keli Chen, Yunzhong Chen

**Affiliations:** 1 College of Pharmacy, Hubei University of Chinese Medicine, Wuhan 430065, China; 2 Hubei Provincial Chinese Medicine-Derived Health Food Engineering Research Center, Wuhan 430065, China

**Keywords:** *Hirudo nipponia*, Orbitrap LC-MS, peptide purification, ACE inhibitor

## Abstract

The renin-angiotensin system (RAS) is the primary pathway for regulating blood pressure in the body, and angiotensin-converting enzymes (ACEs) play a crucial role in it. *Hirudo nipponia* is an invertebrate that contains a variety of active peptides; however, there are no studies on the ACE inhibitory activity of hirudo. In the present study, our aim was to identify the active peptides in hirudo based on active peptide database analysis, unexpectedly filling the gap in hirudo ACE inhibitory activity research. Prep-HPLC was used to separate the part below 3 kD from hirudo. The peptide composition of the isolates was obtained based on Orbitrap LC-MS. The activity of each group of peptides was predicted by the database and the activity was determined by bioassay. Peptides with validation activity were screened through the database. In total, 337 peptides and 18 peptides matching the NCBI leech protein database were identified. All four fractions showed ACE inhibitory activity, and the IC50 was 0.8266, 0.2708, 0.4432, and 0.1764 mg/mL, respectively. Six screened peptides showed good affinity for ACE. This work reveals for the first time that low-molecular-weight peptides from *H. nipponia* have ACE inhibitory activity, which can provide a new explanation for leech treatment of hypertension.

## 1. Introduction

In eastern Asia, mainly in China, there is a widely distributed species of hirudo identified as the *Hirudo nipponia* Whitman, which is the only blood-sucking leech included in the *Pharmacopoeia of the People’s Republic of China*. Hirudo is a well-known Chinese medicine used to promote blood circulation to remove blood stasis [1]. At present, the main active peptides isolated from *H. nipponia* are hirudin, new leech protein-1 (NLP-1), piguamerin, and guamerin. Hirudin is famous worldwide for its powerful antithrombin activity. Natural hirudin is a polypeptide that consists of 64–66 amino acids with a molecular weight of approximately 7000 Da [2]. NLP-1 is an anticoagulant protein with a molecular weight of 13,800 Da [3]. Piguamerin (Mw 5090 Da) is a trypsin inhibitor [4], and guamerin (Mw 6110 Da) is a leukocyte elastase inhibitor [5]. In summary, studies on the active peptides of *H. nipponia* are relatively scarce thus far, and studies on low-molecular-weight peptides are lacking. In addition, the current research has not yet investigated the angiotensin-converting enzyme (ACE) and dipeptidyl peptidase 4 (DPP4) inhibitory activity of leeches. The treatment of hypertension and type 2 diabetes with leeches in traditional Chinese medicine (TCM) is based on the efficacy of leeches in promoting blood circulation and removing blood stasis, which is generally recognized in the theory of TCM [6,7]. In this study, we found the inhibitory effect of ACE and DPP 4 of the leech, which is new evidence that can explain why leeches can treat hypertension and type 2 diabetes.

Angiotensin-converting enzyme, which converts angiotensin I into the potent vasoconstrictor angiotensin II and eliminates the vasodilator effects of bradykinin through its degradation, plays an important role in the control of hypertension [8]. There are many ACE inhibitors on the market, such as captopril, ramipril, and enalapril, which are widely applied in the clinical treatment of hypertension [9]. Consequently, ACE inhibitor substances are used to decrease the blood pressure of hypertensive patients. DPP 4 is a serine protease on the cell surface [10]. DPP 4 can inactivate a variety of bioactive peptides, including glucagon-like peptide-1 (GLP-1) and gastric inhibitory peptide (GIP). DPP 4 inhibitors can inactivate DPP 4 to avoid decomposition of GLP-1 and exert control blood glucose by improving the level of GLP-1 [11]. They are currently one of the main focuses in the treatment of type 2 diabetes [12]. In this study, we analyzed the peptide composition of the four peaks isolated from the 3-kD part of leech by Orbitrap LC-MS, predicted the ACE and DPP4 inhibitory activity of each fraction using activity peptide database analysis, and successfully validated the ACE inhibitory activity. Furthermore, both activities of the leech extract were confirmed. The work involving the peptide activity analysis in this paper is interesting and meaningful because it contributes to the understanding of the mechanism of treating hypertension and type 2 diabetes.

## 2. Materials and Methods

### 2.1. Chemicals and Reagents

The activated partial thromboplastin time (APTT) kit and prothrombin time (PT) kit were purchased from Sun Biotech (Shanghai, China). All chemicals, reagents, and organic solvents of the highest grade available were purchased from Sigma–Aldrich (St. Louis, MO, USA) unless otherwise stated. Leeches were purchased from Jingzhou Minkang Biotechnology Co., Ltd. (Jingzhou, China) and were identified as *H. nipponia.*

### 2.2. Pretreatment and Extraction Method

Fresh leeches (1.6 kg) were washed with water and divided into 4 plates, which were pre-frozen in a cold trap (−45 °C) for 2 h to form ice cubes. The samples were dried in a CTFD-18PT vacuum freeze-dryer (CREATRUST, Qingdao, China) for 72 h (−45 °C, vacuum 60 Pa). The freeze-dried leeches were crushed into a powder and passed through a No. 6 sieve (150 ± 6.6 μm). Leech powder (80 g) was added to 800 mL of pure water and extracted at 4 °C for 2 h, during which it was stirred every 30 min. After extraction, the supernatant was obtained by centrifugation (4 °C, 3000 rpm, 15 min). The residue was extracted again using the same method. The supernatants were combined and concentrated to 600 mL under reduced pressure at 45 °C.

### 2.3. Separation Method

A 3-kD ultrafiltration centrifuge tube (Millipore, Boston, MA, USA) was used, the tube was centrifuged at 3000× *g* rpm for 15 min at 4 °C, and the part passing through the inner tube was collected to obtain extract with a molecular weight below 3 kD, hereinafter referred to as the 3-kD part. The 3-kD part was separated with a Preparative High-Performance Liquid Chromatography 1260 Infinity II (Agilent, Santa Clara, CA, USA). The chromatographic conditions were as follows: the preparative chromatographic column was an Ultimate^®^ AQ-C18 (250 × Í10 mm, 5 µm, Welch Materials, Inc., Shanghai, China). Isocratic elution was used, and phase A was 95% acetic acid aqueous solution (pH 4.0). Phase B was methanol, with a proportion of 5%. The flow velocity was 4 mL/min, and the detection wavelength was 250 nm. The detection of APTT and PT for activity monitoring of the isolation process was carried out.

### 2.4. Orbitrap LC–MS Analysis

The sample was desalted with a 0.1% trifluoroacetic acid aqueous solution by a C18 cartridge (Waters). Then, the peptide was eluted from the column with a 0.1% trifluoroacetic acid aqueous solution containing 50% acetonitrile. After the peptide solution was freeze-dried overnight, 20 μL of 0.1% formic acid was used as a solution for subsequent mass spectrometry.

The detection system was a combination of an Easy-nLC 1200 (P/N LC140, Thermo Fisher Scientific, Waltham, MA, USA) and an Orbitrap Exploris 480 (P/N BRE725533, Thermo Fisher Scientific, Waltham, MA, USA). First, 10 μL of mobile phase A (0.1% formic acid aqueous solution) was used to dissolve and load 5 μL of the sample. The peptide was captured by a trap column (PepMap C18, 100 μm × 2 cm, Thermo Fisher Scientific, Waltham, MA, USA) at a flow rate of 10 μL/min for 3 min. Then, the peptides were separated by gradient elution chromatography on a nanograde analytical column (PepMap C18, 75 μm × 25 cm, Thermo Fisher Scientific, Waltham, MA, USA). The separation gradient was as follows: mobile phase B (0.1% formic acid in acetonitrile) rose from 5% to 30% within 60 min. The chromatographic flow rate was 200 nL/min, and the column temperature was 55 °C. The ion source spray voltage was 2.0 kV, the heating capillary of the mass spectrometer was set to 320 °C, and the data-dependent mode was adopted to automatically switch between MS and MS/MS for acquisition. The full scan MS used an Orbitrap for the primary scan, the scan range was m/z 350–1600, and the resolution was set to 70,000 (m/z 200). The maximum ion introduction time was 50 ms, and the automatic gain control (AGC) was set to 5 × 10^5^. Then, higher energy C-trap dissociation (HCD) was used to fragment the top 15 precursor ions that met the cascade (MS/MS) fragmentation conditions, which were scanned with an Orbitrap. The scan resolution was set to 17,500. The scanning range was automatically controlled according to the mass-to-charge ratio of the precursor ion. The minimum scanning range was fixed at m/z = 110, and the maximum was 2000. The lowest ionic strength value for MS/MS was set to 13,000. In MS/MS, the maximum ion introduction time was 100 ms, the AGC control was set to 2.0 × 10^5^, and the precursor ion selection was set to 1.6 Da. For the MS/MS collection of ions with 2, 3, and 4 charges, dynamic exclusion was set to perform MS/MS once for each precursor ion within 10 s and then exclusion for 40 s with 30% collision energy.

### 2.5. Database Search and Peptide Identification

The raw data used PEAKS Studio 7.0 software for data processing and spectrum analysis. The software can perform de novo sequencing, PEAKS DB (protein identification), multiengine protein characterization, PEAKS PTM (post-translational modification), sequence homology search, and labeled and non-labeled quantification. PEAKS DB software uses the linear discriminative function (LDF) scoring algorithm to judge the quality of the peptide-spectrum match (PSM). The LDF scoring algorithm not only considers the matching of fragment ions and theoretical peaks in the spectrum but also considers many other factors, such as the consistency between the database search peptide and the de novo peptide. The LDF scoring algorithm attempts to achieve two goals: (1) find the correct peptide sequence that best matches each spectrum from the sequence database, and (2) try to distinguish between correct and incorrect matches as much as possible for the entire dataset. When performing de novo sequencing and database retrieval, the parameters were set as follows: NCBI leech protein database, nonenzymatic digestion, tolerance of the primary mass spectrum of 10 ppm, tolerance of the secondary mass spectrum of 0.02 Da, and there was no fixed modification. The variable modification settings were methionine oxidation, N-terminal acetylation, C-terminal amination, and charge settings of +2, +3, and +4. The false-positive rate (FDR) of peptide identification was set to 1%.

### 2.6. Activity Prediction

BIOPEP, a database containing known bioactive peptides [13], was selected to predict the potential activity of the identified peptides. According to the known active polypeptide sequence or fragment, the bioactivity of peptides containing the active sequence or fragment could be predicted. For example, if the determined peptide has a sequence (or fragment) of ACE inhibitory activity, the peptide is assumed to have ACE inhibitory activity. In addition, the active frequency was counted based on the number of ACE inhibitory activity sequences (or fragments) in the identified sequences. For a peptide with a known sequence that contained two sequences (or fragments) with ACE inhibitory activity, the frequency was counted twice. According to the frequency of the potential activity of the peptide sequence identified by each peak, statistical analysis was used to predict the potential activity of each peak.

### 2.7. ACE Inhibition and DPP 4 Inhibition Assay

ACE activity was determined by the amount of 3-hydroxybutylic acid (3HB) generated from 3-hydroxybutylyl-Gly-Gly-Gly with the enzyme method [14]. Based on this principle, a kit produced by DOJINDO was used to test ACE activity. As instructed, enzyme B was dissolved with 2 mL of deionized water to prepare enzyme B solution. Then, 1.5 mL of enzyme B solution was added to enzyme A to prepare the enzyme working solution. Enzyme C and coenzyme were each dissolved with 3 mL of deionized water. Then, 2.8 mL of enzyme C solution and 2.8 mL of coenzyme solution were added to indicator solution to prepare indicator working solution. After preparing two kinds of working solutions, they should be stored in an icebox. According to the instructions, 20 µL of sample solution was added to a sample well in a 96-well plate, and 20 µL of deionized water was added to the blank 1 (positive control) and blank 2 (reagent blank) wells. Next, 20 µL of substrate buffer was added to each well. Then, 20 µL of deionized water was added to the blank 2 well, and 20 µL of enzyme working solution was added to each sample well and blank 1 well. The plate was incubated at 37 °C for 1 h. Next, 200 µL of indicator working solution was added to each well, and the plate was incubated at room temperature for 10 min. The absorbance was read at 450 nm with a microplate reader. Furthermore, 3 replicates for each well were used, and the ACE inhibitory activity (R) was calculated by the following equation:

R (%) = [(A_blank1_ − A_sample_)/(A_blank1_ − A_blank2_)] × 100 (1)

where IC_50_ is defined as the concentration of inhibitor required to inhibit ACE activity by half under certain conditions. IC_50_ was obtained as follows: log[C] was plotted against log(R/(1 − R) (should be a straight line) to obtain the equation Y = aX + b, Y is log(R/(1 − R)), where C is the sample concentration. The value of X was calculated when Y = 0, and X was taken against the number to obtain IC_50_.

For DPP 4 activity, Cayman’s DPP 4 Inhibitor Screening Assay Kit was used. According to the instructions, the final composition of assay buffer was 20 mM Tris-HCl (pH 8.0) containing 100 mM NaCl and 1 mM EDTA. Human Recombination DPP 4 was prepared in 600 µL as instructed. The DPP substrate consisted of a mixture of 120 µL of 5 mM H-Gly-Pro conjugated to amino-methyl-coumarin (AMC) and 2.88 mL of assay buffer solution. After the above preparation, 30 µL of assay buffer, 10 µL of DPP 4, and 10 µL of deionized water were added to 3 initial activity wells. Then, 40 µL of assay buffer was added and 10 µL of deionized water to 3 background wells. Then, 30 µL of assay buffer, 10 μL of DPP 4, and 10 μL of sample solution were added to 3 sample wells. After completing the above steps, the reactions were initiated by adding 50 µL of substrate solution to all the wells. Then, the plate was covered with a plate cover and incubated for 30 min at 37 °C. The plate cover was removed, and the fluorescence was read using an excitation wavelength of 360 nm and an emission wavelength of 460 nm. The DPP 4 inhibitory activity could be calculated by the following equation:

R (%) = [(F_Initial Activity_ − F_Sample_)/(F_Initial Activity_ − F_Background_)] × 100 (2)

### 2.8. ACE Inhibitory Peptide Screening

The analysis was performed using the CALCULATIONS option under the ANALYSIS function in the BIOPEP-UWM database to calculate the score of peptide ACE activity as a way to screen active peptides.

### 2.9. Molecular Docking Study

Human ACE (PDB:1O8A) was obtained from the RCSB PDB database. Peptide structures were generated using Chem3D19.0, and the minimum energy algorithm used to simulate the spatial conformation of the peptides. Molecular docking was accomplished using AutoDock-Vina and the binding energy was used as an indicator to evaluate the optimal conformation of docking [15]. Visualization of the molecular docking was performed using Pymol 4.3.0.

## 3. Results and Discussion

### 3.1. Isolation and Purification

**Figure 1:**
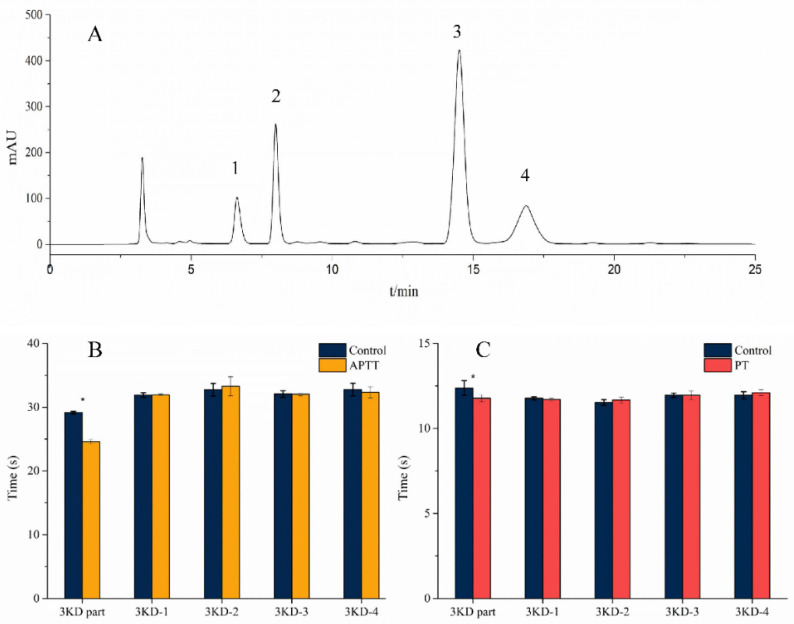
(**A**) Prep-HPLC separation map of the 3-kD part. Labels 1, 2, 3, and 4 are the target peaks. (**B**) The results of the APTT test (*: *p* < 0.05). (**C**) The results of the PT test (*: *p* < 0.05).

A 3-kD ultrafiltration tube was used to obtain the part with a molecular weight below 3 kD. The 3-kD part was tested by APTT (activated partial thromboplastin time) and PT (prothrombin time) to determine the length of coagulation time. As shown in Figure 1B,C, the 3-kD part shortened the APTT and PT times, with statistically significant differences (*p* < 0.05). As shown in Figure 1A, peaks 1, 2, 3, and 4 were purified by prep-HPLC separation, and four peaks were marked as 3KD-1, 3KD-2, 3KD-3, and 3KD-4. The eluent of each peak was collected and freeze-dried, and white powdery crystals were obtained. As shown in Figure 1B,C, 3KD-1, 3KD-2, 3KD-3, and 3KD-4 could not change the timing of APTT and PT, without statistical significance.

Hirudin is the most potent natural thrombin inhibitor known [16]. It was found that the 3-kD part of leech has some shortening of APTT and PT, suggesting that the 3-kD part may have some hemostatic effects, which is an interesting phenomenon, indicating that some hemostatic components may exist in leech. The isolated fractions had no significant effect on the duration of APTT and PT, indicating that the active ingredient was not in these fractions. Active constituents might exist in miscellaneous peaks or other small peaks. The separated peptides were hydrophilic components, so a column that was resistant to the high-water phase should be used. The Ultimate^®^ AQ-C18 column used in this work is a column that can be eluted with 100% water. In this experiment, a good quality separation effect was obtained when the water phase exceeded 95%. Finally, 95% water (pH 4.0) and 5% methanol were selected as the elution system (mobile phase) considering the time and column loss.

### 3.2. Peptide Sequencing and Identification Results

**Figure 2:**
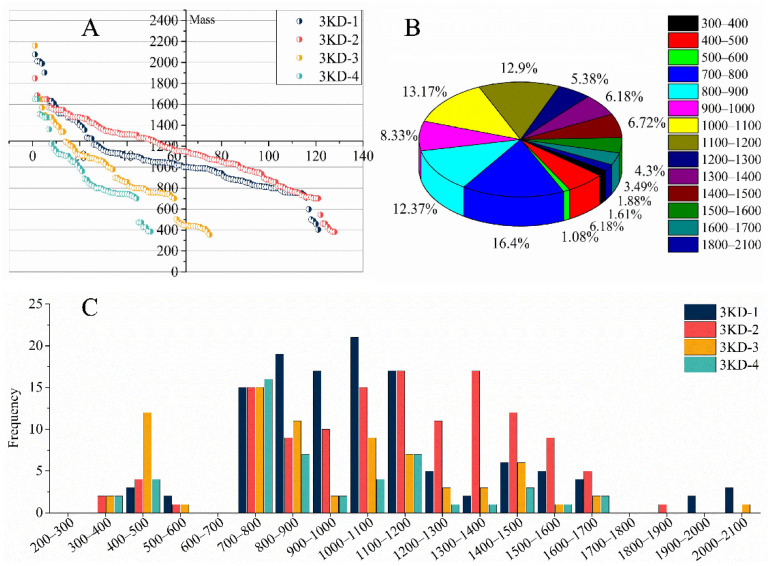
The molecular weight distribution map of each peak peptide. (**A**) The successive distributions of molecular weights of peptides in 3KD-1, 3KD-2, 3KD-3 and 3KD-4 are described, respectively. The horizontal axis is the number of each component peptide and the vertical axis is the distribution of the molecular weight. (**B**) The distribution situation of the percentage of each interval. (**C**) Histograms of the frequency distribution of each interval for the four components.

After mass spectrometry detection, the original data were subjected to de novo sequencing. This sequencing result only selected the results with a credibility of more than 90%. The credibility of 121 peptide sequences in peak 3KD-1 was greater than 90%, and 128 peptide sequences in peak 3KD-2 met the reliability requirements. There were 75 and 50 peptide sequences in peak 3KD-3 and peak 3KD-4, respectively, that met the requirements, and a total of 374 leech peptide sequences (see Annex S1 for detailed results). The sequencing results of each peak were partially duplicated. There were 28 types of repeated peptide sequences, and the total number of repetitions was 65. Excluding the repeated peptide sequences among these samples, there were 337 peptide sequences with a credibility of no less than 90%. Considering the global peptide identifications, the molecular weight ranged between 356 and 2161 Da, with an average molecular weight of 1016 Da (Figure 2A). Overall, 89.25% of the peptides were between 700 and 1700 Da (Figure 2B). The trend of each peak was similar to the overall trend, and most of the peptide composition of each peak was also between 700 and 1700 Da (Figure 2C). It is worth mentioning that no peptides were identified in the molecular weight range of 600–700 and 1700–1800 Da, forming two breaks that disrupt the continuous distribution of molecular weight, which is a strange phenomenon (Figure 2A,C). These data show that the 3-kD ultrafiltration membrane can effectively cut off parts with a molecular weight above 3 kD.

While performing de novo sequencing, protein identification was performed using PEAKS DB software. PEAKS DB uses the LDF (linear discriminative function) scoring algorithm to judge the quality of the peptide-spectrum match (PSM). Generally, a score higher than 20 is considered a successful identification. After analysis, the scores of 18 sequences exceeded 20 (Table 1). In other words, 18 leech peptides were identified based on the existing NCBI leech protein database. There were nine kinds of proteins corresponding to the peptides in Table 1, and the functions of these proteins were searched on UniProt using the accession numbers of these proteins. The functions of the nine kinds of proteins were divided into four categories. These four functions are actin, catalysis, proteolysis, and the constituent proteins of nucleosomes. Similarly, these protein functions were not found to have anticoagulant- and procoagulant-related functions.

### 3.3. Activity Prediction Result

**Figure 3:**
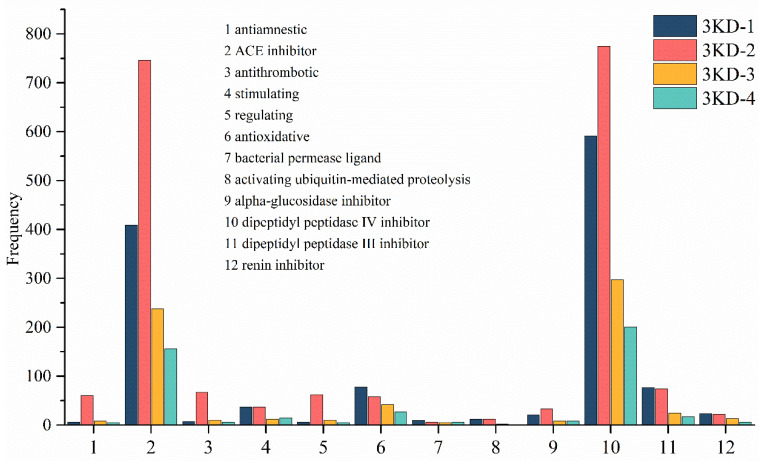
Frequency distribution diagram of the activity prediction results. The vertical axis indicates the frequency of occurrence of the corresponding active fragment, and the horizontal axis indicates the corresponding activity, with each number representing an activity.

Based on the biologically active sequences or fragments contained in the sequences identified in the four peaks by searching the BIOPEP-UWM database, the potential activities of the peptides contained in the four peaks could be inferred. What is interesting is that the first two positions of the potential activity of the four peaks are the same. Among them, DPP 4 inhibitor and ACE inhibitor had an absolute frequency advantage in taking the top two positions. For example, DPP 4 inhibitor ranked first in the potential activity of the components identified by the 3KD-1 peak with a frequency of 591, ACE inhibitor ranked second with a frequency of 409, and antioxidant ranked third with a frequency of 77. As shown in Figure 3, considering the potential activities of the four peaks, the predicted activity was finally selected as DPP 4 inhibitor and ACE inhibitor. ACE inhibitors are also called angiotensin-converting enzyme inhibitors, which is the general term for a class of antihypertensive drugs. In addition, DPP 4 inhibitors and dipeptidyl peptidase 4 inhibitors are a category of drugs meant for the treatment of type II diabetes. The clinical treatment of high blood pressure and type II diabetes with leeches in traditional Chinese medicine may be related to these two potential activities of *H. nipponia.*

It is a worthwhile method to predict the activity of peptides through the active polypeptide database. However, there is a problem with this approach. The method in this study was based on database predictions, and its accuracy was correlated with the amount and structure of the data in the database. The database included 1084 ACE inhibitory peptides and only 432 DPP4 inhibitory peptides. In addition, there were 143 dipeptides among the ACE inhibitory peptides, accounting for 13.19% of the total, and 253 dipeptides among the DPP4 inhibitory peptides, accounting for 58.56%. This raises the question that short fragments (dipeptide) can lead to too high frequency statistics, and the credibility of the prediction results is in doubt. Therefore, the confidence of predicting ACE inhibitory activity is higher than that of DPP4 inhibitory activity, and the prediction of DPP4 inhibitory activity may fail. When using this method to predict peptide activity, the data volume and data structure of the database must be taken into account, and an activity database with a large data volume and low dipeptide percentage will predict the results with higher confidence.

### 3.4. Activity Determination

After DPP4 inhibitory activity determination, the inhibitory rates of 3KD-1, 3KD-2, 3KD-3, and 3KD-4 were 2.43%, 2.04%, 1.31%, and 0.94%, respectively. The four peaks showed almost no DPP4 inhibitory activity. This result is discouraging, but it aptly illustrates the low accuracy of results predicted by activity databases with small amounts of data and a high proportion of dipeptides. However, it is worth mentioning that the leech extract showed good DPP4 inhibitory activity, which was 93.07%, close to the 99.97% of sitagliptin (positive drug). This finding is quite interesting. ACE inhibition activity data were measured for a series of concentration gradients, and the data were processed to derive their equations (Table 2). The IC_50_ values were 0.8266, 0.2708, 0.4432, and 0.1764 mg/mL for each group by equation (Table 2). The IC_50_ value of the leech extract was 0.0653 mg/mL, which showed good activity (Table 2). Among the peas, white pea extract showed the best ACE activity, with IC_50_ values of 0.2–0.3 mg/mL [17]. Compared to white pea extract, 3KD-4 shows comparable activity and leech extract shows superior activity. In the future, leech shown to have superior ACE inhibitory activity will be a point of interest for researchers. In summary, the prediction of ACE inhibitory activity was successful. The comparison with the prediction failure of DPP4 inhibitory activity shows that the accuracy of this method is correlated with the amount of data and the data structure of the database.

Hypertension and diabetes are common diseases that endanger human health nowadays. The renin-angiotensin system (RAS) is the primary pathway for regulating blood pressure in the body [18]. ACE processes the precursor peptide angiotensin I to give angiotensin II, which is a potent vasoconstrictor, and it also destroys the vasodilating properties of a second peptide, bradykinin [19]. DPP-4 inhibitors are important oral antidiabetic agents that in fixed-dose combinations with metformin are widely used. The side effect profile of DPP-4 inhibitors is favorable, there are few treatment-limiting adverse effects, and DPP-4 inhibitors have shown cardiovascular safety [20]. Hirudo has been widely used in the treatment of hypertension and diabetes in TCM due to its efficacy in promoting blood circulation and removing blood stasis. The findings of this study explain, to some extent, the underlying mechanism of the use of leeches in TCM for the treatment of hypertension and type II diabetes, rather than being due to its efficacy in activating blood circulation and resolving blood stasis. In other words, the discovery that leech extract has ACE and DPP4 inhibitory activity provides a new explanation for treating hypertension and diabetes with the traditional Chinese medicine leech.

### 3.5. ACE Inhibitory Peptide Screening Results

The ACE inhibitory activity of each sequence was analyzed through the BIOPEP-UWM database, and the database provides a possibility score when predicting sequence activity. According to the possibility score, these sequences were selected (Table 3). LVVYPWTQR (3402) is an active peptide included in the BIOPEP-UWM database with ACE inhibitor activity [21]. Peptide 3 is highly homologous to LVVYPWTQR, with only one extra Leu at the N-terminal, and its predicted score is 0.30. Therefore, it is more likely to have ACE inhibitory activity. Similarly, peptide 6 has only one more Lys than DGL (9056) [22] at the N-terminal. LLF (7805) [23] and LRP (3543) [24] are the two ACE inhibitory peptides in the database. Peptide 5 consists of seven amino acids and can be seen as LLF (7805) and LRP (3543) (N-terminal) combined and then composed of an Ala at the C-terminus. It is presumed that the N-terminal Leu-Arg-Pro has a positive effect on the affinity of peptide 5 to ACE. Peptide 1 and peptide 2, which have higher scores than peptide 3, are also highly likely to have ACE inhibitory activity. Whether the peptides in Table 3 have ACE activity requires further study to be conclusive.

Generally, the number of amino acid residues of most ACE inhibitory peptides found is 2 to 12, due to the inability of the ACE active site to accommodate large molecular peptides [25]. The six selected peptides have 4–10 amino acid residues and are able to access the active site, which is well reflected by the visualization of the docking results that follows. Studies have shown that Lys, Tyr, Phe, Trp, Pro, and Cys have a positive effect on the activity of ACE inhibitory peptides [26]. The high contents of Lys and Pro in peptide 1 and peptide 2 also indicated that they might have ACE inhibitory activity. Synthetic ACE inhibitors such as Captopril and Lisinopril are available for clinical use, but their side effects remain a cause for concern, such as cough, taste loss, angioneurotic edema, and renal impairment. The peptides derived from natural products are shown to be safer than synthetic drugs [27]. Additionally, peptides have the advantage of multifunctional properties and easy absorption in vivo. These natural peptides have attracted more and more attention from scientists [28]. This groundbreaking work shows that the low-molecular-weight peptides of *H. nipponia* have ACE inhibitory activity. In the future, these natural polypeptides may become a potential functional food for people’s use.

### 3.6. Molecular Docking Results

**Figure 4:**
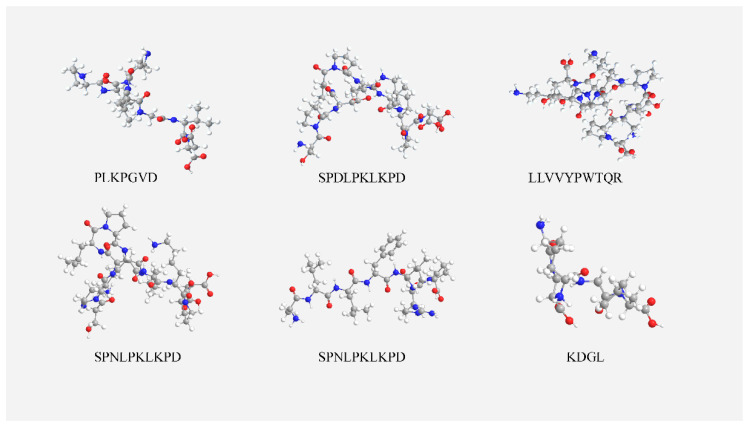
Three-dimensional structure of the peptide. The diagram shows the 3D structure of the peptide. The red spheres are oxygen atoms, the blue spheres are nitrogen atoms, the gray spheres are carbon atoms, and the white spheres are hydrogen atoms.

Figure 4 shows the 3D structure of the peptides simulated with chem3D19.0 as their minimum energy conformation and the 3D structure of the ligand molecule (peptide) is necessary for molecular docking analysis. Molecular docking studies allow prediction of the binding of peptides to ACE and thus analysis of the inhibition mechanism.

The binding energies of the six peptides to ACE were all lower than −4.0 kcal/mol, and the binding energy of peptide 5 was the lowest at −7.2 kcal/mol (Table 4). Usually, the binding energy is below −4.0 kcal/mol and the complex formed by peptide-ACE is relatively stable, and a lower binding energy indicates that the peptide binds more stably to ACE and has better ACE inhibitory activity [29]. The optimal conformation of the peptide is determined mainly by electrostatic and van der Waals interactions [26]. The number of different amino acid residues in the peptide sequence that form hydrogen bonds with ACE is above five (inclusive of five). It was shown that the number of hydrogen bonds likewise determines the affinity of the peptide for binding to ACE [30]. The more hydrogen bonds, the stronger the affinity with ACE.

**Figure 5:**
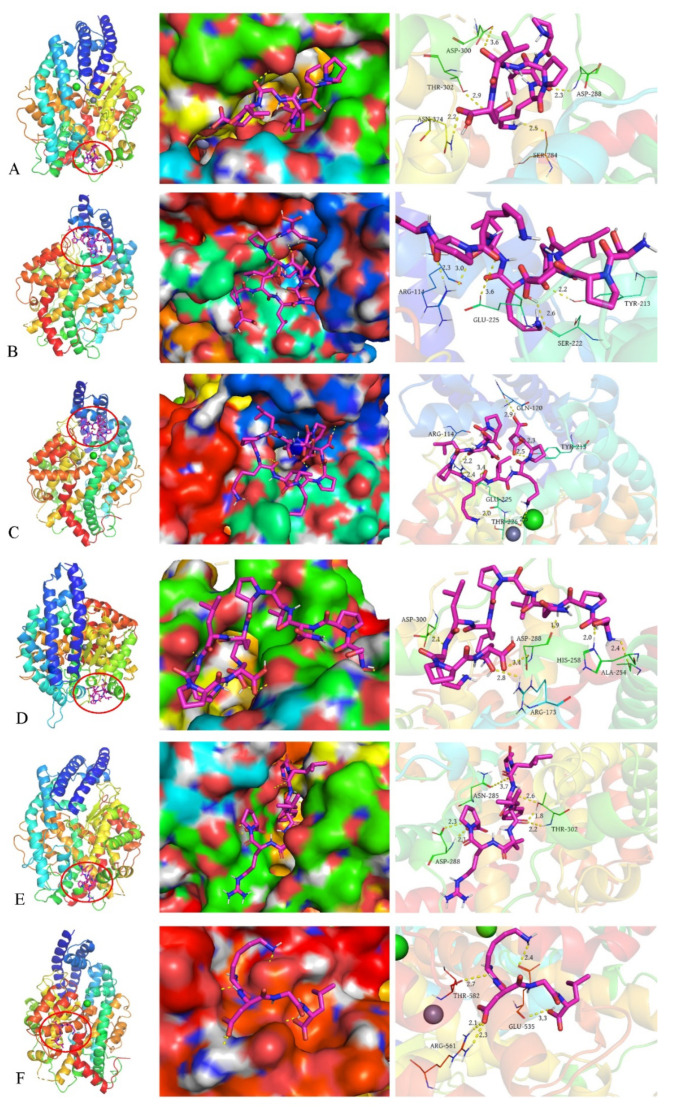
(**A**–**F**) shows the visualization results of the molecular docking of peptides 1–6 with ACE in order. On the left is a cartoon model of the binding of a ligand (peptide) to a receptor (ACE), in which the purple rod-like structure is the ligand (peptide) and the large molecule is the receptor (ACE). In the middle is the local enlargement of the binding of ligand (peptide) and receptor (ACE). The receptor (ACE) is in surface mode to better show the spatial structure, the ligand (peptide) is in stick cartoon mode to better show the interaction between the docked molecules, and the yellow dashed line is the hydrogen bond. On the right is a partial enlarged cartoon diagram of the binding of the ligand (peptide) to the receptor (ACE). The stick structure is the ligand (peptide), the line structure is the amino acid residue and the label next to it is the amino acid number of the receptor (ACE), and the yellow dashed line is the hydrogen bond and the label next to it is the bond length.

Figure 5 demonstrates the visualization results of the six peptides interacting with ACE, where the peptide is the best conformation at the lowest binding energy. The images can help to obtain information about which amino acids of ACE the peptide interacts with and the spatial location of the binding to ACE. Peptide 1 formed five hydrogen bonds with SER-284 (2.5AÅ), ASP-288 (2.3Å), ASP-300 (3.6Å), THR-302 (2.9Å), and ASN-374 (2.2Å) of ACE, respectively (Figure 5A, right). Peptide 1 was embedded in the pore of the ACE spatial conformation (Figure 5A, middle). Peptide 2 formed five hydrogen bonds with ARG-114 (2.3Å,3.0Å), TYR-213 (2.2Å), SER-222 (2.6Å), and GLU-225 (3.6Å) (Figure 5B, right), and peptide 2 was embedded in the pore of the ACE space conformation (Figure 5B, middle). Peptide 3 formed eight hydrogen bonds with ARG-114 (2.2Å, 2.4Å), GLN-120 (2.9Å), TRY-213 (2.3Å, 2.5Å), GLU-225 (2.0Å, 3.4Å), and THR-226 (2.5Å) (Figure 5C, right). Peptide 3 was the peptide that formed the highest number of hydrogen bonds among the six peptides, and likewise embedded in the pore of the ACE spatial conformation (Figure 5C, middle). Peptide 4 was embedded in the pore of the ACE space conformation (Figure 5D, middle), forming six hydrogen bonds with ARG-173 (2.8Å), ALA-254 (2.0Å, 2.4Å), HIS-258 (2.0Å), ASP-288 (1.9Å, 3.4Å), and ASP-300 (2.1Å) (Figure 5D, right). Peptide 5 was embedded in the pore of the ACE spatial conformation (Figure 5E, middle), and formed six hydrogen bonds with ASN-285 (3.7Å), ASP-288 (2.1Å, 2.3Å), and THR-302 (1.8Å, 2.2Å, 2.6Å) (Figure 5E, right). Peptide 6 formed five hydrogen bonds with GLU-535(2.4Å, 3.3Å), ARG-561(2.1Å), and THR-582(2.7Å) (Figure 5F, right). Unlike the above peptides, peptide 6 was only embedded in a depression on the surface of the ACE space conformation (Figure 5F, middle). These peptides interacted with ACE mainly through hydrogen bonds, and no other interactions were found. The visualization docking results show that peptides 1–5 are all embedded in the pore trap of the ACE spatial conformation, and usually this binding situation is more stable. Meanwhile, this binding mode may prevent ACE from binding to its receptor and thus play an inhibitory role. Overall, the screened peptides showed good affinity for ACE.

## 4. Conclusions

The current work describes the research ideas related to the low-molecular-weight peptides of *Hirudo nipponia*, and attempts to determine the possible bioactivity by specific assays and bioinformatics. In this study, an attempt was made to predict the activity of the low-molecular-weight peptides based on the analysis of the active peptide database, and the ACE inhibitory activity of each component was successfully predicted and validated, problems with which were also evident. When using this approach to predict peptide activity, the data volume and data structure of the database must be given attention, and an activity database with a large data volume and low dipeptide proportion predicts more reliable results. Similarly, six peptides with high scores were screened through the ACE inhibitory peptide database, and molecular docking analysis showed that the six peptides had good affinity with ACE, which also increased the reliability of the prediction results. This groundbreaking work shows that the low-molecular-weight peptides of *Hirudo nipponia* have ACE inhibitory activity. On the other hand, the discovery that leech extract has ACE and DPP4 inhibitory activity provides new evidence for treating hypertension and diabetes with the traditional Chinese medicine leech. Hypertension and diabetes are common diseases that endanger human health nowadays. In the future, leech researchers should focus on the activities described in this study, and these natural peptides can be a potential functional food or drug for people’s use.

## Figures and Tables

**Table 1:** Identified peptide information.

Peptide	Mass	Score	Accession	Species	Protein Sources
LVLGGVDVTGPHL	1275.7186	30.02	XP_009029636.1	*Helobdella robusta*	Proteasome subunit beta
VLGGVDVTGPHL	1162.6345	37.43	XP_009029636.1	*Helobdella robusta*	Proteasome subunit beta
YELPDGQVITIGNER	1702.8525	30.36	ESN97261.1	*Helobdella robusta*	hypothetical protein HELRODRAFT_155129
AGFAGDDAPR	975.441	26.17	ESN97261.1	*Helobdella robusta*	hypothetical protein HELRODRAFT_155129
DNIQGITKPAIR	1324.7462	22.92	ESO04417.1	*Helobdella robusta*	Histone H4
DSYVGDEAQSK	1197.5149	24.02	ESN97261.1	*Helobdella robusta*	hypothetical protein HELRODRAFT_155129
GYSFTTTAER	1131.5197	23.87	ESN97261.1	*Helobdella robusta*	hypothetical protein HELRODRAFT_155129
ISGLIYEETR	1179.6135	22.73	ESO04417.1	*Helobdella robusta*	Histone H4
NVINGGSHAGNKL	1279.6633	23.26	XP_009015226	*Helobdella robusta*	Phosphopyruvate hydratase
SYELPDGQVITIGNER	1789.8846	31.66	ESN97261.1	*Helobdella robusta*	hypothetical protein HELRODRAFT_155129
TVTAM (+15.99) DVVYALK	1325.6901	27.09	ESO04417.1	*Helobdella robusta*	Histone H4
YSNRVVD	851.4137	23.05	ESO03354.1	*Helobdella robusta*	Glyceraldehyde-3-phosphate dehydrogenase
YSNRVVDL	964.4978	22.95	ESO03354.1	*Helobdella robusta*	Glyceraldehyde-3-phosphate dehydrogenase
ESTLHLVLR	1066.6135	25.55	XP_009027975.1	*Helobdella robusta*	hypothetical protein HELRODRAFT_193870
GYSFTTTAER	1131.5197	23.66	ESN97261.1	*Helobdella robusta*	hypothetical protein HELRODRAFT_155129
TVTAMDVVYALK	1309.6952	35.92	ESO04417.1	*Helobdella robusta*	Histone H4
PSIVGRPR	880.5242	25.26	ESN97261.1	*Helobdella robusta*	hypothetical protein HELRODRAFT_155129
PSLVGRPR	880.5242	25.26	ABC60435.1	*Hirudo medicinalis*	cytoplasmic actin

**Table 2:** IC_50_ values and calculation equation of ACE inhibitory activity in different groups.

Group	IC_50_ (mg/mL)	Equation	r Value
3KD-1	0.8266	y = 0.5950x − 1.7358	0.9976
3KD-2	0.2708	y = 0.8372x − 2.0366	0.9951
3KD-3	0.4432	y = 0.944x − 2.4984	0.9938
3KD-4	0.1764	y = 1.0675x − 2.3982	0.9972
Leech extract	0.0653	y = 1.4515x − 2.6347	0.9971

**Table 3:** Potential ACE active peptides.

Number	Peptide	Mass	Score	ACE Inhibitor Peptide (ID ^1^)
1	PLKPGVD	724.4119	0.45	PL (7513), LKP (7549), GV (7608), PG (7625), KP (7810)
2	SPDLPKLKPD	1108.6128	0.34	DLP (7542), LKP (7549), KL (7693), KP (7810)
3	LLVVYPWTQR	1273.7183	0.30	LVVYPWTQR (3402), YPWTQR (3403), VY (3492), VYP (3505), YP (3666), TQ (7834), VVYPW (7750), VVYPWTQ (9277), LVVYPWTQ (9278)
4	SPNLPKLKPD	1108.6128	0.19	LKP (7549), KL (7693), KP (7810)
5	ALLFLRP	828.5222	0.15	LRP (3543), LF (3551), RP (7582), LLF (7807), LR (9213)
6	KDGL	431.2380	0.14	GL (7599), DG (7681), DGL (9056)

^1^ The number in parentheses in the rightmost column is the ID of the polypeptide in the BIOPEP-UWM database.

**Table 4:** Molecular docking results.

Number	Peptide	Binding Energy (kcal/mol)	Hydrogen Bond
1	PLKPGVD	−6.1	5
2	SPDLPKLKPD	−6.3	5
3	LLVVYPWTQR	−5.8	8
4	SPNLPKLKPD	−5.8	6
5	ALLFLRP	−7.2	6
6	KDGL	−5.6	5

## Data Availability

Not applicable.

## Supplementary Materials

The following supporting information can be downloaded at: https://www.mdpi.com/article/10.3390/molecules27175421/s1. Annex S1 is available as Supplementary Materials uploaded with the article.
